# Identifying Facilitators and Obstacles in Piloting Dementia Initiatives Within a Living Lab Approach: Systematic Review

**DOI:** 10.2196/77752

**Published:** 2026-03-31

**Authors:** Sara Alves, Natália Duarte, Teodora Figueiredo, Luis Midao, Maria João Bessa, Olalla Rodriguéz, José Fidalgo, Irene Fernandez, Alba Felpete, Maxi Rodriguéz, Juan Carlos Bernardéz, David Facal, Joana Carrilho, Elísio Costa

**Affiliations:** 1 CIDIFAD – Centro de Investigação, Diagnóstico, Formação e Acompanhamento das Demências, Santa Casa de Misericórdia de Riba D'Ave, Travessa Conde de Riba de Ave, Braga, 4765-267, Portugal, 351 252900900; 2 ICBAS - School of Medicine and Biomedical Sciences, RISE-Health, Porto, Portugal; 3 Biochemistry Lab, Faculty of Pharmacy of the University of Porto, RISE-Health, Porto, Portugal; 4 Competences Centre on Active and Healthy Ageing (Porto4Ageing), Faculty of Pharmacy of the University of Porto, Porto, Portugal; 5 UPTEC-Science and Technology Park of University of Porto, Porto, Portugal; 6 Galician Health Service (SERGAS), ACIS - Galician Agency of Knowledge in Health, Santiago de Compostela, Spain; 7 Department of Developmental Psychology, University of Santiago de Compostela, Santiago de Compostela, Spain; 8 Instituto de Investigación Sanitaria de Santiago de Compostela (Health Research Institute of Santiago de Compostela, IDIS), Santiago de Compostela, Spain; 9 Institute of Psychology, University of Santiago de Compostela, Santiago de Compostela, Spain; 10 AFAGA Alzheimer - Asociación de Familiares de Enfermos de Alzheimer y otras Demencias de Galicia, Vigo, Spain

**Keywords:** cognitive impairment, digital health, innovation, quadruple helix approach, user-centered approach, real-world application

## Abstract

**Background:**

Dementia affects more than 55 million people worldwide, and its progressive cognitive decline creates substantial challenges for intervention testing and real-world implementation. Living Labs (LLs) have become increasingly relevant for piloting interventions in dementia care, offering real-world environments for cocreation and iterative testing. However, operational, ethical, and governance challenges can hinder the effective implementation of dementia-focused initiatives.

**Objective:**

This study aimed to identify and synthesize the facilitators and challenges encountered when piloting dementia-related initiatives within an LL approach.

**Methods:**

A systematic review was conducted in PubMed, Web of Science, Scopus, and EBSCOhost from database inception to January 31, 2024. Studies were eligible if they reported the piloting, implementation, or evaluation of dementia-related initiatives in an LL context. A narrative synthesis was performed using content analysis for initial coding and thematic analysis for theme development. The systematic review was conducted in accordance with PRISMA (Preferred Reporting Items for Systematic Reviews and Meta-Analyses) guidelines.

**Results:**

The 15 included studies were mostly conducted in European countries. Two major themes emerged, namely (1) organizational and operational issues and (2) ethical and legal considerations. Within these, 4 key operational subthemes were identified: engaging people living with dementia (end users), research design and evaluation methods, co-design and testing phases, and LL governance. Ethical and legal considerations covered informed consent procedures and regulatory aspects. Across studies, the most frequently reported facilitators involved familiar environments, stakeholder collaboration, iterative evaluation, and flexible co-design processes. Barriers commonly included fluctuating cognitive symptoms, limited digital literacy, inconsistent stakeholder engagement, time and resource constraints, and fragmented regulatory guidance.

**Conclusions:**

Piloting dementia initiatives in LLs requires methodological adaptability, strong governance structures, and ethical procedures tailored to cognitive impairment. These findings provide actionable guidance for researchers, practitioners, industry partners, and policymakers seeking to develop sustainable, user-centered LLs that support innovation in dementia care.

## Introduction

Globally, it is estimated that 55 million people live with dementia, with this number projected to increase to 152 million by 2050 [1]. Dementia is a progressive neurodegenerative disorder that compromises memory, behavior, personality, judgment, attention, spatial awareness, language, abstract thought, and other executive functions [2]. These cognitive impairments significantly reduce individuals’ ability to function socially, occupationally, and independently [3]. The growing impact of dementia presents a major public health challenge due to its extensive burden on people living with the disease, families, caregivers, health care professionals, and society as a whole [1,4].

Efforts to improve dementia care have led to the development of innovative approaches that extend beyond traditional clinical and technological interventions. While advances such as artificial intelligence–assisted diagnostics, smart home monitoring, and virtual reality therapies have shown potential, many fail to adequately address the lived experience of people living with dementia [5,6]. This highlights the need for more inclusive, user-centered strategies that embed people living with dementia and their caregivers throughout the innovation process.

In this context, Living Labs (LLs) have emerged as valuable frameworks for cocreating and testing solutions in real-life environments [7]. LLs are user-centered, real-world settings that integrate research and innovation through co-design, iterative testing, and multistakeholder collaboration, following a quadruple-helix approach that connects academia, industry, government, and civil society [8].

Their strength lies in their capacity to foster collaboration between researchers, health care providers, patients, and caregivers, bringing scientific knowledge into everyday practice and allowing continuous feedback loops for refinement and validation [9,10]. However, the application of LLs in dementia care is particularly complex. The multifaceted nature of dementia, ethical considerations related to vulnerable participants, and the logistical and governance demands of maintaining multiactor environments all present significant challenges [10-13].

Previous studies have explored some of these aspects, identifying both enabling and constraining factors in operating LLs in health care. Some of the reported barriers include challenges related to the disease’s impact on end users’ participation [14,15], such as difficulties in decision-making and evaluation processes [11,12]; securing adequate funding [10]; addressing ethical concerns related to vulnerable populations [16,17]; and managing logistical issues to create realistic yet controlled environments [18]. Conversely, facilitators of successful LLs include strong stakeholder collaboration, robust technological infrastructure, and well-established ethical frameworks [19,20], which enhance the effectiveness and impact of these initiatives. Nonetheless, existing evidence remains fragmented and largely focused on single-case experiences or technological pilots. A comprehensive synthesis of facilitators and barriers specific to dementia-related LLs is still lacking.

Given the increasing prevalence of dementia and the need to optimize innovation processes in real-world contexts, this systematic review aims to identify and synthesize the facilitators and barriers encountered when piloting dementia-related initiatives within an LL framework. By consolidating available evidence, this study seeks to inform the design, implementation, and governance of sustainable, ethically sound, and user-centered LLs that can enhance innovation in dementia care.

Therefore, this review aims to systematically identify and synthesize the facilitators and barriers encountered when piloting dementia-related initiatives within LL environments.

## Methods

### Search Strategy

A systematic search was conducted in 4 databases for relevant literature: PubMed, Web of Science, Scopus, and EBSCOhost, from database inception to January 31, 2024. Keywords included MeSH (Medical Subject Headings) terms and synonyms as follows: “living lab” AND “dementia” AND “barrier” OR “obstacles” OR “facilitators” OR “enablers.” Details of the search strategy, including specific search strings and filters used, are available in Multimedia Appendix 1.

### Inclusion and Exclusion Criteria

Studies were included if they met all of the following criteria, namely (1) reporting findings within an LL approach (with consideration for multimethod approaches, user engagement, multistakeholder participation, real-life settings, and cocreation); (2) piloting initiatives related to dementia or cognitive decline; (3) randomized controlled trials, observational studies, qualitative studies, mixed methods studies, intervention studies, implementation science studies, case studies, and systematic reviews or meta-analyses or scoping reviews; and (4) publications written in English, Spanish, or Portuguese.

Studies were excluded if they met any of the following criteria, namely (1) those not addressing the topic of barriers and facilitators of piloting initiatives; (2) those describing or analyzing the technical parameters of a digital solution; (3) those comprising samples with cognitive problems not directly related to dementia or degenerative cognitive impairment (eg, aphasia); and (4) study protocols, theoretical papers, conceptual papers, and position papers.

### Study Selection and Data Extraction

After retrieving manuscripts, duplicates were removed using EndNote software (v21; Clarivate Analytics). Subsequently, 2 authors (SA and ND) independently assessed the titles and abstracts of all potentially relevant articles. The full texts of these articles were then meticulously reviewed to determine final eligibility based on predefined inclusion and exclusion criteria. Any discrepancies were resolved through consensus with a third reviewer (TF), who conducted an independent analysis of such manuscripts. Additionally, reference lists of the included articles were screened to identify supplementary relevant studies.

To ensure consistency in data extraction, an Excel spreadsheet (Office 21, v.2405; Microsoft Corporation) was created to document key study information, including author details, publication year, country, study design, sample size, participant type (eg, people with dementia, professionals, caregivers, and family members), and a brief description of the piloted initiatives. Extracted data also included key facilitators and barriers reported in each study.

### Data Synthesis

A narrative synthesis was conducted in accordance with the PRISMA (Preferred Reporting Items for Systematic Reviews and Meta-Analyses) 2020 guidelines. The synthesis followed two sequential analytical stages:

1. Content analysis was first applied to systematically code all reported facilitators and barriers across studies, identifying recurring concepts and relationships.
2. Thematic analysis was subsequently used to cluster and refine these codes into overarching themes and subthemes [21].

Two reviewers (SA and ND) independently performed the coding using NVivo software (v.14.23.3; Lumivero LLC). Regular meetings were held to discuss coding discrepancies and reach consensus, with a third reviewer (TF) available for arbitration when needed.

Following an iterative review of the extracted data and initial coding, two overarching themes were identified: (1) organizational and operational issues and (2) ethical and legal considerations. Each of these themes was further divided into subtopics that encompassed both barriers and facilitators [22]. Themes and subtopics were refined and organized into a final framework of facilitators and barriers.

A formal risk of bias assessment was not conducted, as the included studies primarily focused on methodological and participatory approaches for engaging people living with dementia under an LL approach rather than interventional studies. However, to ensure transparency, a completed PRISMA checklist is provided as supplementary material in Checklist 1. This review was registered in PROSPERO (CDR42024514481).

## Results

### Overview

**Figure 1.**
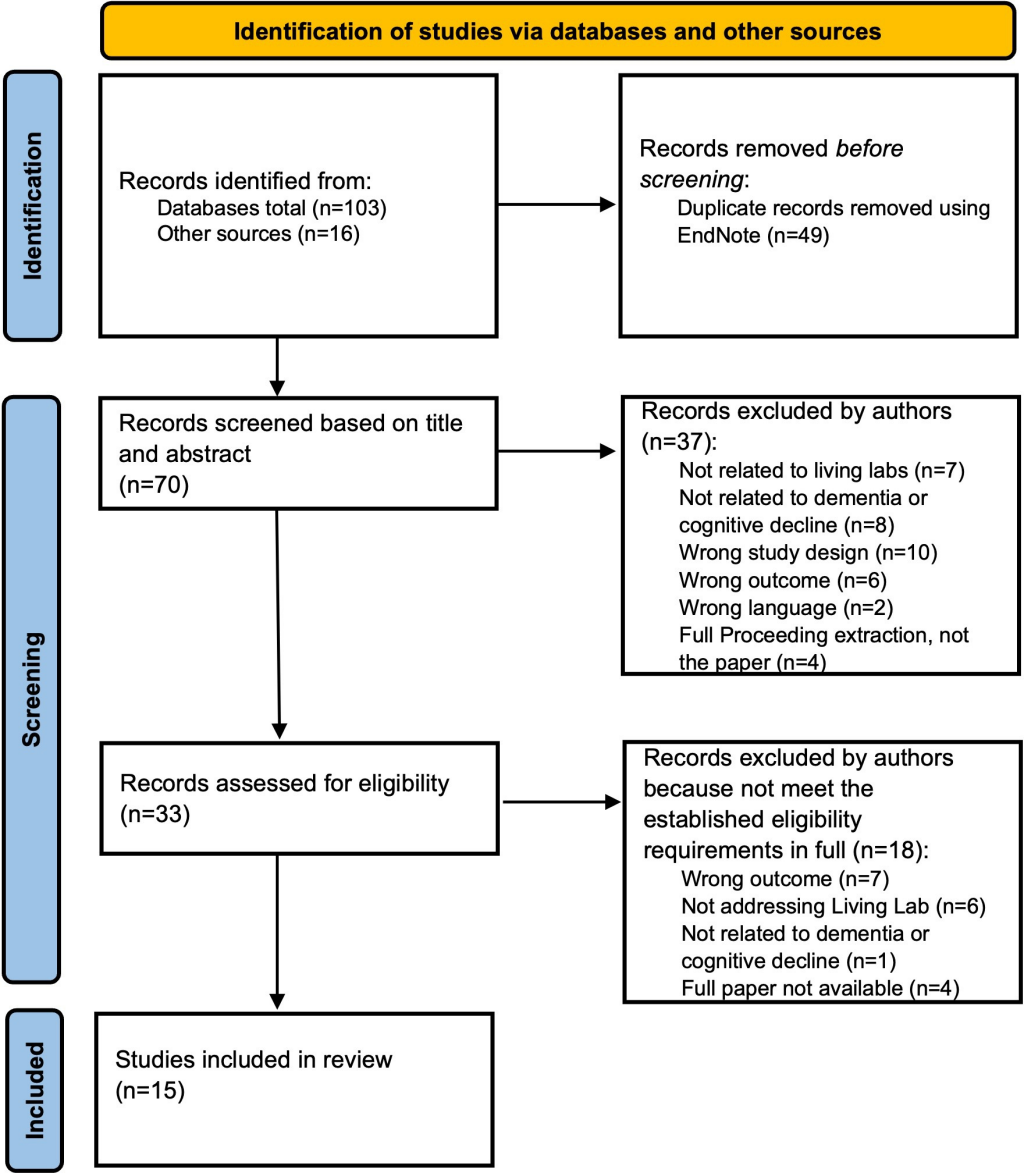
PRISMA (Preferred Reporting Items for Systematic Reviews and Meta-Analyses) flowchart of the included studies.

The initial search resulted in a total of 119 publications. After duplicate removal and full-text analysis, 15 met the inclusion criteria (Figure 1).

Findings were synthesized narratively through content and thematic analysis, according to the predefined analytical framework. Two overarching themes emerged, which were (1) organizational and operational issues and (2) ethical and legal considerations, each comprising several subthemes that captured both facilitators and barriers. Of the 15 studies [18,23-36], engaging people living with dementia was the most frequently reported subtheme (14 studies) [18,23-31,33-36], followed by research design and evaluation methods (12 studies) [23-31,33-36], co-design and testing phases (12 studies) [23-31,34-36], and LL governance (10 studies) [18,23,26,28-31,33-35].

The studies were mainly qualitative (n=10) [18,23-25,28-32,35] and mixed methods (n=5) [26,27,33,34,36] (Multimedia Appendix 2). The reviewed articles were all from Europe, with the exception of 1 article from Canada, that is, Pigot and Giroux [23]. Facilitators and barriers were presented below by themes and subtopics. Multimedia Appendix 2 provides an overview of the included studies, while Table 1 summarizes facilitators and barriers by theme.

**Table 1.** Summary of facilitators and barriers by theme.

Theme	Subthemes	Most frequent facilitators	Most frequent barriers
Organizational and operational issues	Engaging people living with dementia Research design and evaluation methods Co-design and testing phases Living Lab governance	Friendly atmosphere, familiar settings, caregiver involvement, and clear communication Mixed methods approaches, visual aids, iterative feedback, and involvement of key informants Real-life testing, pilot testing with healthy adults, training sessions, and flexible procedures Quadruple-helix collaboration, shared vision, transparency, and regular meetings	Digital literacy limitations, cognitive fluctuations, perceived stigma, and privacy concerns Low adherence to questionnaires, positive-response bias, inconsistent data, and difficulty demonstrating real-world benefits Time constraints, technical malfunctions, interruptions, and recruitment difficulties Funding shortages, administrative burden, communication gaps, and divergent stakeholder priorities
Ethical and legal considerations	Informed consent procedures Regulatory aspects	Rolling consent, adapted forms, and oral and written explanations Alignment with ethical standards, involvement of gatekeepers, and certification	Difficulty assessing capacity, limitations of one-time consent, and unclear evidence on audiovisual tools Fragmented regulations, data-protection challenges, and lack of standardized contracts

### Theme 1: Organizational and Operational Issues

Within the theme of organizational and operational aspects, 14 [18,23-31,33-36] out of 15 studies [18,23-36] identified various facilitators and barriers in piloting dementia initiatives using the LL approach. Four subthemes were identified within this theme: engaging people living with dementia, research design and evaluation methods, co-design and testing phases, and LL governance.

### Engaging People Living With Dementia (End Users)

#### Facilitators

Fourteen studies emphasized strategies that promoted meaningful involvement of people living with dementia [18,23-31,33-36]. Common facilitators included a relaxed atmosphere and assisting with transportation [24].

The use of native language, careful pacing of information, and ensuring users were well-informed, using accessible language, were also mentioned [26,27]. Other studies highlighted the importance of a friendly atmosphere (eg, welcome participants with coffee or tea; coffee breaks; and not to dive into the product immediately, but starting by making informal contact first), familiar settings (eg, real-life conditions), and involving familiar people and care professionals to make end users feel safe and understood [18,23,28-31]. Recognizing the real-world benefits of initiatives and managing participants’ expectations were described as crucial aspects for fostering engagement [18,27,34]. Another aspect that emerged was the possibility of offering rewards to increase users’ willingness to participate in the initiatives [35].

#### Barriers

Several challenges related to engagement in LL activities were noted in 8 of the reviewed studies [26,28,29,31,34-36]. Some challenges include the participation of users with limited technical skills, which made it harder for them to fully participate. Additionally, not all user suggestions could be implemented due to technical or practical constraints (eg, development stage of the prototype), which sometimes caused frustration or a sense of exclusion because users felt their contributions were not fully considered [31,34]. Furthermore, negative perceptions of the solution, such as seeing it as stigmatizing or too complex to use, can lead to reduced confidence or reluctance to participate, ultimately hindering the engagement of people living with dementia [31,35,36]. Privacy concerns also limited participation, as participants reported reluctance to allow sensor monitoring in private home areas [36]. Other barriers include reluctance to express criticism or fear negative consequences, which can affect their willingness to provide feedback [28].

### Research Design and Evaluation Methods

#### Facilitators

Mixed methods approaches to evaluate studies under an LL framework were endorsed in 12 studies [23-28,30,31,33-36], highlighting its potential to provide a comprehensive understanding of the LL approach, as well as real-world and user experiences. Qualitative methods, such as semistructured interviews and focus groups, effectively captured the experiences of people living with dementia. The use of visual aids, key informants, nonintrusive evaluation instruments [31,33], and postsession reflections [24,31] was recommended to enhance engagement. Additionally, a user-centered research design that emphasizes cocreation and continuous stakeholder feedback was highlighted in 5 studies [26,28,30,31,33] as essential for developing relevant dementia interventions.

#### Barriers

Challenges in research design and evaluation methods were noted in 10 [23-25,27,28,30,31,33-35] of the 15 reviewed studies [18,23-36], and included low adherence to questionnaires, bias toward positive feedback, and inconsistent data collection, as well as issues like team changes and lack of diversity among participants that may limit study findings [35]. Other barriers included challenges in demonstrating the real-world benefits of initiatives [28,33,34], and ensuring sustained involvement throughout the project lifecycle [33].

### Co-Design and Testing Phases

#### Facilitators

One widely recognized facilitator, mentioned in 12 [23-31,34-36] out of 15 studies [18,23-36], is the importance of integrating co-design and testing phases into daily activities and real-life settings. Other aspects included clear staff roles and environments tailored to the users’ needs, using flexible facilities, nonintrusive methods, and adapting testing conditions for users’ difficulties [23,25,26,28-31,34]. Conducting pilots with healthy older adults before involving people living with dementia was suggested as an effective strategy to identify potential issues early and adjust the testing process accordingly [24,30,34], helping to prevent confusion for end users. Implementing training sessions prior to deploying solutions [24,30] was also recognized as a valuable strategy.

#### Barriers

Challenges related to the co-design and testing phases were noted in 8 studies [23,26,28,30,31,34-36]. These included time constraints, budget limitations, and technical difficulties [26,34,35]. Additionally, the fluctuating symptoms of dementia require highly adaptable solutions, which can be difficult to find [31]. As an example, it was found that users’ attention and motor fluctuations affected interaction with digital prototypes, and it was observed that caregivers had to adapt testing sessions daily to participants’ cognitive variability [30,31].

Other studies pointed to the difficulties of tests in real-world settings [36], such as interruptions during home interventions, potential performance blur between user and relatives, and conflicts between user and researcher agendas [23].

### LL Governance

#### Facilitators

Governance facilitators were highlighted by 10 studies [23,26,28-31,33-36]. Quadruple helix approach through the collaboration of academia, industry, government, and civil society, nurturing a culture of openness and team belonging, was described as an important aspect to ensure a smooth implementation of the innovation process [23,28,29]. Other facilitators highly mentioned included regular meetings and stakeholders’ communication, clearly defined roles, and the development of roadmaps, which contribute to the LL transparency.

#### Barriers

Governance challenges were identified in 7 studies [18,26,28,31,33-35]. The main identified barriers included funding, time-consuming processes, defining a shared vision among stakeholders, balancing stakeholder interests, maintaining communication across all stakeholders, and managing complex administrative tasks.

### Theme 2: Ethical and Legal Considerations

Eight [18,24,26,27,29-32] out of 15 studies [18,23-36] identified ethical and legal facilitators and barriers relevant to a dementia-focused LL approach. These findings were categorized into 2 main topics: informed consent procedures and regulatory aspects. While all studies discussed facilitators and/or barriers related to informed consent procedures, only 4 of them [18,27,29,31] addressed regulatory aspects (refer to Multimedia Appendix 2 for further details).

### Informed Consent Procedures

#### Facilitators

“Process consent” (ie, ongoing seeking permission from participants throughout the study duration) or “rolling consent” (ie, seeking permission from participants as new phases or new activities are introduced) was advocated in 5 [18,26,27,29,32] studies with recommendations to monitor participant reactions and regularly remind them of their option to withdraw [31]. The importance of using both oral and written consent formats was also highlighted [29,32]. Additionally, sending consent forms in advance and assessing participants’ capacity to consent were emphasized as key ethical practices [30,32]. Obtaining consent from both people living with dementia and their representatives, using adapted communication strategies, and considering innovative approaches, such as interactive consents (ie, a dynamic and ongoing process of seeking and reaffirming consent from participants) were also noted in some studies [31,32].

#### Barriers

Challenges related to the obtainment of informed consents were noted in 3 [26,27,32] studies. They include difficulties with one-time consent agreements [26,27] or the complexity of assessing consent capability due to fluctuating cognitive abilities [32]. It was also noted that there is still a lack of evidence that validates the benefits of using audio-visual aids in the consent process [32], which may limit their adoption.

### Regulatory Aspects

#### Facilitators

Regulatory considerations, such as aligning prototypes and research methods with ethical standards and regulatory aspects, were discussed in 2 studies [27,31]. These studies emphasized the involvement of gatekeepers (ie, individuals or organizations who ensure the protection and well-being of people living with dementia, commonly including care institutions, relatives, and health professionals) or regulatory bodies and consistent regulations of recognized authorities (eg, European Network of Living Labs [ENoLL]) to foster collaboration while safeguarding participants’ interests.

#### Barriers

Regulatory barriers were highlighted in 3 studies [18,29,31]. These included fragmented regulations across regions, lack of specific guidelines for cross-country research, absence of standardized contractual models, and challenges in managing sensitive data and intellectual property (mainly due to the collaborative nature and iterative process of LL, which often blurs the lines between roles).

## Discussion

### Principal Findings

This systematic review identified key facilitators and barriers that influence the successful piloting of dementia initiatives within an LL approach. Two overarching themes emerged, namely “Organizational and Operational Issues” and “Ethical and Legal Considerations,” each encompassing a series of recurrent subthemes across the 15 included studies [18,23-36]. Overall, the findings show that LLs hold significant potential to support innovation in dementia care, but their effectiveness depends heavily on adapting methodological, ethical, and governance procedures to the specific needs and vulnerabilities of people living with dementia.

Consistent with previous literature, the studies highlighted that engaging people living with dementia requires approaches that accommodate fluctuating cognitive abilities, promote familiarity, and reduce cognitive burden [19,37,38]. Facilitators such as friendly atmospheres, familiar settings, accessible communication formats, and the involvement of caregivers and professionals emerged as crucial to fostering meaningful engagement. These findings illustrate the inherent complexity of piloting solutions with this population, reinforcing the need for tailored, adaptable, and iterative strategies. They also underscore the importance of ethical awareness and safeguarding the rights and well-being of people living with dementia while ensuring that their perspectives are integrated throughout the co-design process.

Based on the analysis of barriers and facilitators, 3 core aspects emerge as central to understanding the functioning of LLs in dementia care: the target population features, the LL ecosystem**,** and the LL framework gaps.

### The Target Population Features

This dimension encompasses the unique needs and challenges faced by people living with dementia, including aspects that influence their participation, engagement, and adherence to the cocreation and testing processes.

The studies showed that users’ heterogeneous and fluctuating cognitive symptoms create significant challenges from the earliest stages of the innovation process. These fluctuations affect communication, emotional stability, and the ability to engage with tasks, requiring highly flexible planning and individualized support [39-42].

Aspects related to communication processes (eg, accessible communication, native language, written and oral information, visual aids, regular reminders, and managing information load), flexible and adapted assessment methods and iteration phases, and familiar settings and people, and consistent team members are essential considerations to handle the unpredictable nature of dementia. Addressing these challenges often requires involving proxies (eg, caregivers and relatives), which extends the time needed for the process and may increase communication challenges and stakeholders’ differing perspectives. These consequences ultimately influence project governance, sustainability, and the overall pace of development within LLs [38,43,44].

Another key aspect concerns the perceived benefits of the initiatives. Several studies emphasized that people living with dementia and their caregivers are more likely to accept and use a technology or intervention when they perceive clear, immediate value [18,28,31,33,36]. This aligns with previous research that documented that technology is either not accepted at all or is quickly abandoned when users do not perceive an immediate benefit [45-47]. Ensuring simple, intuitive interfaces and avoiding stigmatizing features (eg, making solutions too discreet or discouraging their use within social circles) is therefore essential. Failing to address these factors may lead to user frustration and a refusal to engage or discontinuation of use.

### LL Ecosystem

At the ecosystem level, the findings highlight the importance of establishing strong communication flows and working on nurturing the strategic alliances. While the quadruple helix approach provides a solid basis for multiactor collaboration, many studies emphasize the need to expand beyond traditional stakeholders to include society, the media, and local communities [48-52]. This could create more opportunities for digital innovation, diversify funding sources, and contribute to the sustainability of the LL. Sustainability emerged as a recurring challenge, with several studies identifying resource instability and limited long-term planning as barriers to continuity [18,31,33].

Some authors have proposed a “mixed-economy” approach, where both state and private funding support the innovation process in social care [18,45,53]. This approach could be particularly useful in promoting the sustainability of LLs focused on innovation in dementia care.

Another key point is the establishment of a clear goal and shared vision for the LL. The studies reviewed strongly emphasize that LL success requires a clear goal that integrates reliability, real-world benefit, and the well-being of the LL team members. Communication is central to achieving this. It allows collaboration, builds trust, and fosters a sense of belonging. Attention must be given to all stages, from preimplementation, ongoing activities, and postimplementation. Notably, the postimplementation phase appears particularly neglected, with limited evidence on long-term monitoring, communication, and impact assessment (eg, reports, communication, and long-term impact assessment) [54-56]. This gap raises concerns about whether solutions remain relevant and functional after initial deployment, given the progressive nature of dementia [19,43,57].

In dementia care, especially with digital solutions, ongoing monitoring is crucial. Since dementia is a progressive neurodegenerative disorder, adherence to digital solutions will be affected by the disease’s progression. Understanding these factors is vital to ensure that digital solutions effectively support the quality of life of people living with dementia and their caregivers, and therefore, should be a central concern of LL activity. Without this focus, LL risks introducing products that do not meet their intended purpose or do not provide real benefit.

### LL Framework Gaps

As a relatively new approach, LLs still lack a well-defined regulatory structure. The absence of standardized guidelines leads to inconsistencies across countries and sectors, creating challenges in legal, ethical, and operational domains. This includes heterogeneity in data protection requirements, intellectual property procedures, and ethical oversight mechanisms. The limited dissemination of national dementia strategies, with less than a quarter of countries having them in place, further complicates the development of consistent standards, particularly in these populations [58].

Ethical challenges represent a core concern, particularly regarding informed consent and data protection [16,45,59,60]. Cognitive decline complicates the assessment of consent capacity and increases the need for sensitive, adaptive consent procedures. Innovative approaches such as “rolling consent” or interactive consent forms, ensuring that participants are continuously informed of their rights and can withdraw from studies at any time, fostering trust and ensuring participant safety. Privacy concerns, especially in data-rich environments such as LLs, where monitoring and data collection occur in real-life settings (including homes, hospitals, and nursing homes), further complicate the ethical management of these activities. This complexity underscores the need for robust ethical and legal regulations for dementia-focused LLs.

Mechanisms such as ethical review boards, certification authorities (eg, ENoLL), and standard contractual models can support the establishment of responsible LL operations. Strengthening these structures is critical to fostering trust, enhancing participation, and enabling LLs to function effectively in dementia-focused contexts.

### Recommendations for Practice

Based on the results of this study, we have established some recommendations for piloting dementia initiatives (or on cognitive impairment) using an LL approach, providing a practical overview of the findings. The proposed guidelines are organized according to the topics identified in this systematic review (Table 2).

By considering these specific recommendations, LLs can improve their functioning and tackle common challenges in piloting dementia initiatives. Also, these guidelines can contribute to improving practices and to fostering sustainable and efficient innovation processes. By covering a broad range of aspects that include not only user and community involvement, but also research, governance, and ethical and regulatory considerations, these guidelines provide practical guidance for academia (research), policymakers, industry, and health care agents. For example, integrating iterative user feedback and ethical reviews can help tailor initiatives more effectively; furthermore, establishing clear metrics for evaluating the initiatives’ impact will be crucial for assessing their effectiveness. At the ultimate end, these guidelines have the potential to enhance the impact of the LL approach in the dementia field, leading to improved health outcomes and quality of life of those affected, ensuring that innovation processes meet users’ needs effectively.

**Table 2.** Recommendations for operating a dementia Living Lab (based on the categories of results).

Category	Recommendations
Engaging people living with dementia (end users)	Develop engagement strategies tailored to the characteristics of people living with dementia by focusing on their needs, creating a relaxed atmosphere, using accessible language, and involving close persons, including both relatives and care professionals. Consider technological difficulties of users, offering simplified digital solutions along with close technical support to enhance usability and acceptability. Monitor frequently the users’ feedback and experience to avoid feelings of frustration and to ensure users feel respected and understood. Prioritize solutions that respect privacy and avoid being intrusive or stigmatizing. Keep users well-informed by clearly presenting the potential real-world benefits of the solution, its limitations, and setting realistic expectations.
Evaluation methods	Privilege mixed methods approaches, particularly semistructured interviews and focus groups, that help people living with dementia to better express their perspectives. Use alternative communication methods, such as visual aids, and involve close family members or caregivers to enhance communication. Consider cognitive limitations when selecting tools by using observation and cognitive-specific instruments. Ensure staff consistency throughout the process and assure appropriate training on evaluation assessments. Foster friendly and nonintrusive evaluation environments that are flexible and prevent positive biased feedback. Incorporate a postreflection session to gather more in-depth feedback from users.
Co-design and testing phases	Integrate co-design and testing phases in real-life settings to make the process more natural and less disruptive. Ensure that testing environments are familiar, avoiding the need for users to adapt to unfamiliar situations. Conducting pilots with healthy older adults before involving people living with dementia to ensure the prototype functions well and is user-friendly. Plan the co-design and testing phases with extended time and resources to accommodate potential fluctuations in dementia symptoms. Consider the technical requirements of the solutions, including font size, format, battery life, menu design, icons, and audio-visual and written content. Ensure staff consistency and provide appropriate training both for staff and users. Tailor solutions to users’ needs and conduct regular checkpoints to ensure they remain effective.
Research design	Consider co-creation and exploratory approaches to develop effective solutions for dementia. Prioritize study designs that use a user-centered approach, combining empirical and naturalistic methods. Encourage collaboration among stakeholders to create a research design that meets the needs of all partners and maximizes the relevance and impact of the outcomes. Clearly communicate research procedures to all stakeholders throughout the project lifecycle, especially those with varying levels of involvement, and establish defined research protocols specifically for dementia interventions.
LL^a^ governance	Adopt a quadruple helix approach by involving academia, industry, government, and civil society. Establish a shared vision early in the project and balance the interests of all stakeholders to ensure smooth implementation. Foster a sense of team belonging through regular communication, clearly defined roles, and the development of roadmaps. Implement efficient management and administrative strategies to streamline processes and enhance project execution, while avoiding unnecessary bureaucratic procedures. Focus on building a broad network of local, national, and international partnerships and seek certification from recognized authorities (eg, ENoLL^b^). Develop a sustainable business model to secure adequate funding and ensure the long-term sustainability of Living Lab. Raise awareness of Living Lab initiatives by implementing a robust communication plan that encourages community involvement and engagement. Consider establishing multidisciplinary boards, including financial, administrative, and scientific experts, to provide comprehensive oversight and guidance. Integrate a long-term perspective in innovation processes, including postimplementation monitoring. Regularly report on the impact of Living Lab initiatives, clearly identifying their benefits, with a particular focus on the real-world impact for users.
Informed consent procedures	Prioritize “process consent” or “rolling consent,” regularly reminding participants of their rights and option to withdraw. Observe any reactions that may indicate reluctance to participate. Adopt consent-friendly procedures, such as using adapted consent forms and sending informed consent documents in advance for review. Use both oral and written consent formats, explore innovative methods such as interactive informed consent to enhance engagement. Consider using tools to assess the capability to consent, and when necessary, obtain consent from both the individual and their representative.
Regulatory aspects	Involve gatekeepers or regulatory bodies to ensure that research practices adhere to ethical standards and regulatory requirements. Adopt regulatory procedures by standardizing contractual models and establishing clear guidelines for managing intellectual property and data privacy. Advocate for enhanced regulatory procedures at both national and international levels to promote harmonized practices and facilitate cross-country activities.

a LL: Living Lab.

b ENoLL: European Network of Living Labs.

### Limitations

This systematic review is subject to certain limitations. First, despite a comprehensive search strategy across 4 major databases, relevant studies may have been overlooked due to language restrictions (English, Spanish, and Portuguese), and the still-emerging nature of research on dementia-focused LLs. These factors may have contributed to publication bias and limited the diversity of included contexts.

Another limitation of this study pertains to the thematic analysis of the findings, which is susceptible to the influence of the authors’ backgrounds and understanding of the topic. To strengthen trustworthiness, 2 authors independently coded and discussed emerging categories, and interpretations were refined through consensus and alignment with existing literature and the review’s guiding question.

A formal risk of bias assessment was not conducted, as this review focused on synthesizing facilitators and barriers rather than evaluating intervention effects. While this may limit comparability across studies, a structured appraisal was not applicable given the descriptive and exploratory nature of the evidence. Future reviews should incorporate formal bias assessments when suitable tools for qualitative and mixed methods LL research become available.

Finally, the geographical distribution of the publications is predominantly from European Union countries. Out of 15 studies included [18,23-36], only 1 was outside Europe, which could limit the generalization of the findings and reduce the applicability of the conclusions to other contexts. Further research is needed to explore how national dementia strategies, funding mechanisms, and societal attitudes influence LL implementation beyond European contexts.

### Conclusion

In conclusion, this systematic review identifies key aspects for successfully piloting dementia initiatives within an LL framework. A comprehensive and strategically planned approach is essential to effectively address real-world needs. It encompasses a user-centered design that accounts for the cognitive limitations of people living with dementia and balances the interests of stakeholders. Equally important is the development of robust governance models that foster collaboration, ensure smooth communication, promote extensive networking, and secure consistent and long-term funding, all supported by clear ethical guidelines and regulatory standards. By establishing equilibrium among stakeholders’ interests and ensuring adaptability, LLs can serve as effective platforms for driving innovation in dementia care.

## Supplementary material

10.2196/77752 Multimedia Appendix 1 Search terms.

10.2196/77752 Multimedia Appendix 2 Included studies.

10.2196/77752 Checklist 1 PRISMA checklist.

## Abbreviations

ENoLL: European Network of Living Labs
LL: Living Lab
MeSH: Medical Subject Headings
PRISMA: Preferred Reporting Items for Systematic Reviews and Meta-Analyses
