# Supplementary material for: Identifying Facilitators and Obstacles in Piloting Dementia Initiatives Within a Living Lab Approach: Systematic Review
Source: JMIR Aging. 2026 Mar 31;9:e77752. doi: 10.2196/77752 (PMC13037770; doi:10.2196/77752)
Supplement: Multimedia Appendix 1 [file aging-v9-e77752-s001.docx]

| **Supplementary Table 1:**  **Search terms, Databases search strategies** | | |
| --- | --- | --- |
| **Database** | **Search terms** | **Results (number of articles, n)** |
| PubMed | "living lab*"[Title/Abstract] AND "dement*"[Title/Abstract] OR "amentia*"[Title/Abstract] OR "cognitive impair*"[Title/Abstract] OR "cognitive defect"[Title/Abstract] OR "alzheimer*"[Title/Abstract] OR "amnesia"[Title/Abstract] OR "neurocognitive disorder*"[Title/Abstract] OR "cognition disorder*"[Title/Abstract] OR "traumatic psychose*"[Title/Abstract] OR "Korsakoff"[Title/Abstract] OR "Huntington"[Title/Abstract] OR "Lewy Body"[Title/Abstract] OR "delirium"[Title/Abstract] OR "aphasia*"[Title/Abstract] OR "apraxia*"[Title/Abstract]) AND ("barrier*"[Title/Abstract] OR "obstacl*"[Title/Abstract] OR "challeng*"[Title/Abstract] OR "imped*"[Title/Abstract] OR "obstruct"[Title/Abstract] OR "hindrance"[Title/Abstract] OR "interfer*"[Title/Abstract] OR "promot*"[Title/Abstract] OR "facilitat*"[Title/Abstract] OR "implement*"[Title/Abstract] OR "installation"[Title/Abstract] OR "motivat*"[Title/Abstract] OR "driver*"[Title/Abstract] OR "stimulat*"[Title/Abstract] OR "determinant*"[Title/Abstract] OR "support*"[Title/Abstract] OR "aid"[Title/Abstract] OR "aiding"[Title/Abstract] OR "assist*"[Title/Abstract] OR "help*"[Title/Abstract] OR "enabl*"[Title/Abstract] OR "tool*"[Title/Abstract]) | n=16 |
| Web of Science | ((TS=(“living lab*”)) AND TS=(Dement* OR Amentia* OR cognitive impair* OR “cognitive defect" OR Alzheimer* OR amnesia OR neurocognitive disorder* OR cognition disorder* OR traumatic psychose* OR Korsakoff OR Huntington OR "Lewy Body" OR delirium OR Aphasia* OR Apraxia* )) AND TS=(Barrier* OR obstacl* OR challeng* OR imped* OR obstruct OR hindrance OR interfer* OR promot* OR facilitat* OR implement* OR installation OR motivat* OR driver* OR stimulat* OR determinant* OR support* OR aid OR aiding OR assist* OR help* OR enabl* OR tool* ) | n=31 |
| Scopus | TITLE-ABS-KEY ( ( "living lab*" ) AND ( dement* OR amentia* OR "cognitive impair*" OR "cognitive defect" OR alzheimer* OR amnesia OR "neurocognitive disorder*" OR "cognition disorder*" OR "traumatic psychose*" OR korsakoff OR huntington OR "Lewy Body" OR delirium ) AND ( barrier* OR obstacl* OR challeng* OR imped* OR obstruct OR hindrance OR interfer* OR promot* OR facilitat* OR implement* OR installation OR motivat* OR driver OR stimulat* OR determinant* OR support* OR aid OR aiding OR assist* OR help* OR enabl* OR tool ) ) | n=43 |
| EBSCOhost | AB “living lab*” AND AB ( Dement* OR Amentia* OR cognitive impair* OR “cognitive defect" OR Alzheimer* OR amnesia OR neurocognitive disorder* OR cognition disorder* OR traumatic psychose* OR Korsakoff OR Huntington OR "Lewy Body" OR delirium OR Aphasia* OR Apraxia* ) AND AB ( Barrier* OR obstacl* OR challeng* OR imped* OR obstruct OR hindrance OR interfer* OR promot* OR facilitat* OR implement* OR installation OR motivat* OR driver* OR stimulat* OR determinant* OR support* OR aid OR aiding OR assist* OR help* OR enabl* OR tool* )  +  TI “living lab*” AND TI ( Dement* OR Amentia* OR cognitive impair* OR “cognitive defect" OR Alzheimer* OR amnesia OR neurocognitive disorder* OR cognition disorder* OR traumatic psychose* OR Korsakoff OR Huntington OR "Lewy Body" OR delirium OR Aphasia* OR Apraxia* ) AND TI ( Barrier* OR obstacl* OR challeng* OR imped* OR obstruct OR hindrance OR interfer* OR promot* OR facilitat* OR implement* OR installation OR motivat* OR driver* OR stimulat* OR determinant* OR support* OR aid OR aiding OR assist* OR help* OR enabl* OR tool* ) | n=13 (11+2) |
| Total | | n=103 |
| Total after removing duplicates | | n=54 |
