# Supplementary material for: Identifying Facilitators and Obstacles in Piloting Dementia Initiatives Within a Living Lab Approach: Systematic Review
Source: JMIR Aging. 2026 Mar 31;9:e77752. doi: 10.2196/77752 (PMC13037770; doi:10.2196/77752)
Supplement: Multimedia Appendix 2 [file aging-v9-e77752-s002.doc]

**Table S1.** Barriers and facilitators in piloting dementia initiatives within Living Labs (N=15).

| **Author (year); country** |  | **Study design** | **Type of participant (n^a^)** | **Piloted initiative** | **Facilitators** | **Barriers** |
| --- | --- | --- | --- | --- | --- | --- |
| Boman et al (2014) [24]; Sweden |  | Qualitative case study design | - People living with dementia (n=4) - Relatives (n=4) | Videophone mock-up | - Engaging people living with dementia - Assisting people living with dementia with transportation to sessions - Creating a relaxed atmosphere - Research design and evaluation methods - Ensuring effective participation from people living with dementia, not just relatives - Postsessions reflections with the team - Co-design and testing phases - Clear and defined staff roles - Home-like testing environment - Good functioning of the prototype - Conducting a pilot with healthy older adults to identify issues - Providing verbal system support or a demonstration - Allowing extra time for user assimilation - Adapt testing to users' difficulties | - Research design and evaluation methods - - Risk of biased positive feedback   Co-design and testing phases   - - Proper testing conditions (eg, lighting) |
| Brankaert et al 2017 [25]; Netherlands |  | Explorative in-context study | - Professionals (not mentioned) | Qwiek.up | - Engaging people living with dementia - Participants’ and relatives’ attitude and expectations - Research design and evaluation methods - Cocreation perspective enabled the interpretation of results - Explorative approach - Privileging focus group approach - Co-design and testing phases - Embedded in daily activities - Flexibility to use the solution | - Research design and evaluation methods - Low adherence to completing questionnaires |
| Brankaert et al 2015 [26]; Netherlands |  | Multiple case study | - Case study 1: n=4 - Case study 2: n=12 - Case study 3: n=10 | Case study 1: reminder system Case study 2: daylight lamp Case study 3: mobile interface | Engaging people living with dementia   - Users well informed - Pacing and information load during the first contact   Research Design & Evaluation Methods   - Reflection sessions with users   Co-design and testing phases   - Adapt testing to users' difficulties - Embedded in daily activities - Consistent team members throughout tests - Intermediate participants' checkpoints (by phone) - Extended time for testing - Connected to user's needs   LL governance   - Quadruple helix approach - Expanded stakeholder network Informed consent procedures - Privileging "process consent" or "rolling consent" | Engaging people living with dementia   - Some users wanted to continue, but it wasn't always possible - Users could not keep the products   Co-design and testing phases   - Many objectives in the introduction session - To forget previously given information due to memory loss   LL governance   - Late stakeholder involvement in the study phase - Communication across all stakeholders Informed consent procedures - One time-agreement |
| Brankaert et al 2014 [27]; Netherlands |  | Explorative in-context study | - People living with dementia and their counterparts (n=9 dyads) | Smartphone | Engaging people living with dementia  - Native language  Research Design & Evaluation Methods   - Subjective component - Freedom to skip questions - Attractive questionnaires with varied questions - Develop a method to directly involve people living with dementia   Co-design and testing phases   - Long battery of equipment and simple menus   Informed consent procedures   - Privileging "process consent" or "rolling consent"   Regulatory aspects   - Gatekeepers to monitor research and dementia participation | Research Design & Evaluation Methods   - Low adherence to completing questionnaires Informed consent procedures - One-time agreement |
| Bühler et al 2023 [28]; Germany |  | Qualitative study | - Group 1: nursing care professionals (n=13) - Group 2: people with dementia, their relatives, and their representatives (n=14) - Group 3: care and nursing researchers (n=8) | N/A^c^ | Engaging people living with dementia   - Accessible language - Friendly atmosphere - Physical meetings - Recognition of the real-world benefit - Interaction with others - To communicate tasks clearly but without pressure   Research Design & Evaluation Methods   - Alignment with care practice needs   Co-design and testing phases   - Familiar people, formats, and occasions - Not to pressure people living with dementia to adapt to unfamiliar situations   LL governance   - Culture of openness and team belonging - Perceived practical benefits - Quadruple helix approach - Define research-care collaboration through specific tasks - Regular joint meetings - Offering tangible opportunities to get involved | Engaging people living with dementia   - Privileging non-presential contacts (eg, videocalls) - To fear negative consequences - To feel inhibited to express criticism   Research Design & Evaluation Methods   - Communication of research procedures   Co-design and testing phases   - Declined adaptability - Professionals' tendency to offer too much care   LL governance   - Time-consuming for defining a shared vision - Management challenges - Use titles |
| Callari et al 2019 [29]; United Kingdom |  | Qualitative study | - Researchers (n=7) - Experts (n=5) - Staff (n=6) - Residents (n=6) - Family members (n=2) | N/A | Engaging people living with dementia   - Accessible language - Close persons’ involvement - Keep family members and carers informed   Co-design and testing phases   - Clear and defined staff roles - Embedded in real-life settings - Ensure information is in various formats (eg visual and audio) - Considering content, size of font, and format   LL governance  - Multiple stakeholders’ involvement   - Culture of openness and team belonging - Staff ensure smooth projects and link researchers with residents - Pre-project collaborative work and roadmaps - Quadruple helix approach - Building trust and operational transparency   Informed consent procedures   - Oral and written consent - Privileging “process consent” or “rolling consent” - Training on obtaining consent appropriately - To repeat information on an iterative basis - Communicating continuously the option to opt out or withdraw | Engaging people living with dementia   - Family availability for non-critical matters - Ambivalence about research participation - Maintaining user interest and participation - Risk of withdrawal during the LL project - Lack feedback or follow-through   Regulatory aspects   - Challenges in user data and privacy protection - Lack of guidance and ethics for LL research programs - Variation across countries, organizations, and disciplines affects cross-country LLs |
| Geerts et al 2020 [30]; Netherlands |  | Qualitative study | - People living with dementia and their informal caregivers (N^b^=18) |  | Engaging people living with dementia   - Friendly atmosphere - Privilege familiar settings such as home - Pacing and information load during the first contact - Sense of usefulness and being respected - Care professionals' involvement - Close persons’ involvement - Explain study limitations: time, budget, and technical constraints - Interaction with others - Concrete ideas for easier feedback   Research Design & Evaluation Methods   - Involvement of key informants - Visual aids (ie, photographs) may be useful - Use of semi-structured interviews - Privileging open-ended questions - Iterative cycles evaluation - Privileging focus group, open-ended interviews and opinions - Consider the participation of both, people living with dementia and informal caregivers - Use different materials in focus groups and interviews   Co-design and testing phases   - Check the criteria and explain the study procedures - Embedded in real-life settings - Conducting a pilot with healthy older adults to identify issues - Ensuring people living with dementia have or can acquire the necessary skills   LL Governance   - Quadruple helix approach   Informed consent procedures   - Written consent - To send the informed consent in advance for analysis | Engaging people living with dementia   - Not all user inputs can be implemented - Pure co-design can be challenging for people living with dementia due to its abstract nature   Research Design & Evaluation Methods   - The use of closed-ended questions   Co-design and testing phases   - Processing time of information by users - Project iteration is limited by time constraints, budget and technical possibilities |
| Pigot and Giroux 2015 [23]; Canada |  | Lessons learned | N/A | N/A | Engaging people living with dementia   - Close persons’ involvement - Care professionals' involvement - Set rules for avoiding overwhelming residents with requests - Discrete solutions to avoid stigmatization   Research Design & Evaluation Methods   - Involvement of key informants - Realized under realistic scenarios   Co-design and testing phases   - Assistance adapted to users' difficulties - Adapt testing to users' difficulties - Good functioning of prototype - Making explicit instructions - To do frequent summaries to avoid memory overload - Conducting training sessions before deploying the solution   LLs’ Governance   - Continuous communication and roadmaps | Research Design & Evaluation Methods   - Data collection in natural settings risks disrupting habits and memory - Data reliability issues in uncontrolled environments   Co-design and testing phases   - Home interventions linked to anxiety - Real-life testing may be “uncontrolled” (e.g. interruptions) - Tasks may blur between user and relatives - Conflicting agendas between users and the research team |
| Pino et al 2014 [31]; France |  | Multiple case study | Case study 1: SESIN and Hadagio telehealth system Case study 2: Blue Linea and the BlueGard GPS Location Bracelet | N/M^d^ | Engaging people living with dementia   - Sense of usefulness and being respected - Improvement of self-esteem and social inclusion - High confidence to use digital solutions - Using real-life data to adapt communication strategies - Testing initiative must be connected to the user's needs - Use of different communication strategies (verbal and non-verbal) - Close persons’ involvement   Research Design & Evaluation Methods   - Observation instruments - Privileging open-ended interviews - Using multidimensional assessment instruments - Use of mixed-methods approaches - Collecting user feedback post-launch solution - User-centered approach - Use of mixed-methods approaches   Co-design and testing phases   - Flexible facilities for in-situ observations - Non-intrusive methods - Good functioning of the prototype - Users may participate either occasionally or in regular user group - Testing sessions as recreational activities - To deeply profile users (eg, abilities, limitations, attitudes, lifestyle)   LLs’ Governance   - Certification by recognized authorities - Quadruple helix approach - Get support from recognized entities that raise LL awareness - Multidisciplinary teams including "administrative and financial board" as well as “scientific board” - Build a local, national, and international network - Training staff on dementia care - Implement a community-focused dissemination plan   Informed consent procedures   - Both agreement from people living with dementia and representative - Adapted consent forms - Use of different communication strategies - Observe reactions that may suggest reluctance to participate   Regulatory aspects   - Prototypes and methods adjusted to meet ethical standards | Engaging people living with dementia   - Limited digital experience - Poor perceived value of the solution - Low quality of prototype - Perceiving the solution as stigmatizing - Lack of awareness about AT products and services   Research Design & Evaluation Methods   - Costly and time consuming   Co-design and testing phases   - Fluctuating symptoms require highly adaptive and customizable solutions - Perceptual and motor difficulties   LLs’ Governance   - To balance the interests of the different stakeholders - Funding - The definition of the role of private stakeholders - Scarce dissemination campaigns - Tackle challenges related with large cohorts, big data, complex measures, and substantial resources   Regulatory aspects   - Fragmentation among entities and regional variations in laws and regulation - Legal and political framework for LLs |
| Sacco et al 2021 [32]; France |  | Narrative review | N/A | N/A | Informed consent procedures   - Novel formats: interactive informed consents may be appealing - Both agreement from people living with dementia and representative - To stop the research process if divergent opinions occur between people living with dementia and representative - Use an instrument to assess the capability to consent - Privileging “process consent” or “rolling consent” | Informed consent procedures   - Unclear benefits of audio-visual support - Consenting difficulties due to fluctuating symptoms |
| Smith et al 2022 [33]; United Kingdom |  | Case study | N/A | N/A | Engaging people living with dementia   - Friendly atmosphere - Manage feelings of frustration - Close persons’ involvement - Care professionals’ involvement   Research Design & Evaluation Methods   - Ensuring effective participation from people living with dementia - Integrate empirical and naturalistic methods - Methods defined with quadruple helix partners   LLs’ Governance   - Quadruple helix approach - Regularly meetings with all stakeholders - Select initiatives that offer real-world benefits | Research Design & Evaluation Methods   - Challenge of demonstrating real-world benefits LLs’ Governance - Sustainability - Time-consuming processes |
| Snaphaan et al 2011 [34]; Netherlands |  | Case study | People living with dementia (n=6) Researchers (n=6) Healthcare professionals (n=2) Developers (n=4) | Serious game | Engaging people living with dementia   - Sense of usefulness - Interaction with others - Friendly atmosphere - Explain study limitations - Close persons’ involvement - Care professionals’ involvement   Research Design & Evaluation Methods   - Use of mixed-methods approaches - Many moments for data collection - Staff training for harmonized procedures   Co-design and testing phases   - Conducting a pilot with healthy older adults to identify issues - Embedded in real-life settings - Privilege home as testing settings   LLs’ Governance   - Continuous communication and roadmaps - Quadruple helix approach - Updated reports on piloted initiatives for stakeholders | Engaging people living with dementia   - Not all user inputs can be implemented   Research Design & Evaluation Methods   - Retrospective measures   Co-design and testing phases   - Time constraints, budget and technical possibilities - Login difficulties (difficult passwords)   LLs’ Governance   - Decisions often take longer |
| Toso et al 2023 [35]; Netherlands |  | Lessons learned | N/A | N/A | Engaging people living with dementia   - Closing email from Living Lab with findings and acknowledgment - Gifts or discounts to reward user participation - Pacing and information load during the first contact - Friendly atmosphere - Close persons’ involvement   Research Design & Evaluation Methods   - Visual aids (ie, photographs) may be useful - In-person interview with the user - Use of mixed-methods approaches - Involvement of key informants - Many moments for data collection - Staff training for harmonized procedures - Core protocol addressing dementia interventions   Co-design and testing phases   - Flexibility to use the solution - Conducting training sessions before deploying the solution - Intermediate participants' checkpoints   LLs’ Governance   - Involve people living with dementia as experts - Close collaboration with research and care organizations - Defining waves/calls - Multidisciplinary Supervision Committee - Cooperation agreement outlining the testing setup | Engaging people living with dementia   - Solution and supporting documents in a foreign language - Family availability for non-critical matters - Solutions considered too complex were not easily accepted - Perceiving the solution as stigmatizing - Delays in assistance caused frustration and drop-outs   Research Design & Evaluation Methods   - Difficulty involving diverse participants across initiatives can bias the study's findings on adoption or outcomes - Risk of biased positive feedback - Team member changes   Co-design and testing phases   - Real-world settings lacking the product's technical requirements - Recruitment issues due to people living with dementia’s AT skills or product requirements - Participant recruitment as a time-consuming task - Random selection of participants - Care professionals lack time of time - Users’ anxiety due to product failures   LLs’ Governance   - Collaboration with external organizations - To balance the interests of the different stakeholders |
| Vasileiadis et al 2016 [36]; Greece |  | Case study | People with mild cognitive impairment (n=4) | Smart-home sensors and RGB-D cameras | Engaging people living with dementia   - Ensure the users' privacy - Non-monitoring private areas - Recognition of the real-world benefit   Research Design and Evaluation Methods   - Realized under realistic scenarios - Observation instruments   Co-design and testing phases   - Conducting training sessions before deploying the solution | Engaging people living with dementia   - Perceiving the solution as stigmatizing - Reluctance to participate in testing within their social circle   Co-design and testing phases   - Major home changes |
| Verloo et al 2021 [18]; Switzerland |  | Scoping Review | N/A | N/A | Engaging people living with dementia   - Close persons’ involvement - Recognition of the real-world benefit   LLs’ Governance   - Multiple stakeholders’ involvement - Quadruple helix approach   Informed consent procedures   - Privileging "process consent" or "rolling consent" | LLs’ Governance   - Users’ involvement due to disease - Funding   Regulatory aspects   - Manage intellectual property - Lack of a contractual or agreement model - Legal and political framework for LLs |
